# Motley Crew: Overview of the Currently Available Phage Diversity

**DOI:** 10.3389/fmicb.2020.579452

**Published:** 2020-10-29

**Authors:** Nikita Zrelovs, Andris Dislers, Andris Kazaks

**Affiliations:** Latvian Biomedical Research and Study Centre, Riga, Latvia

**Keywords:** complete genome, phage diversity, bioinformatics, genome overview, bacteriophage

## Abstract

The first complete genome that was sequenced at the beginning of the sequencing era was that of a phage, since then researchers throughout the world have been steadily describing and publishing genomes from a wide array of phages, uncovering the secrets of the most abundant and diverse biological entities known to man. Currently, we are experiencing an unprecedented rate of novel bacteriophage discovery, which can be seen from the fact that the amount of complete bacteriophage genome entries in public sequence repositories has more than doubled in the past 3 years and is steadily growing without showing any sign of slowing down. The amount of publicly available phage genome-related data can be overwhelming and has been summarized in literature before but quickly becomes out of date. Thus, the aim of this paper is to briefly outline currently available phage diversity data for public acknowledgment that could possibly encourage and stimulate future “depth” studies of particular groups of phages or their gene products.

## Introduction

The first completely sequenced genome of a bacteriophage (MS2) was reported as early as 1976 (Fiers et al., 1976), however, it is due to the recent advancements in the throughput of sequencing technology and the growth of their affordability along with the development of omics approaches that have really pushed the limits of many studies in a range of biological fields, including the phage diversity research field (Goodwin et al., 2016). Previously deemed as “the dark matter of the biological world,” given their enormous diversity, bacteriophages and their genomes still withhold many secrets even after more than a century since their discovery. The unraveling of which might be of great benefit not only to our understanding of life, but also to the advancement of various scientific fields. It was previously noted that many of the widely used phage-derived products in the field of molecular biology originated from a strikingly small collection of phages that were studied in detail (Schoenfeld et al., 2010; Hatfull, 2015). Today we can see that phage research, in general, is taking two major directions – “depth” (how well do we know phages) and “breadth” (how many phages do we know) – that are intertwined and complement each other to the point that one cannot be taken into account without the other. While the first one is trying to deduce or alter the functions of the phage gene products of interest, the latter steadily provides new data for phage diversity, widening the known phage pangenome from which candidates for future functional-structural studies can be mined (Hayes et al., 2017).

## Situation as of Today

As of June 2, 2020 the NCBI Nucleotide database, when filtered to show only complete bacteriophage genomes, lists 13,132 entries that represent individual phage complete genomes as stated by the respective submission authors (Clark et al., 2016). A total of 265 records had an “unverified” comment by the GenBank staff (229 out of these 265 sequences had no respective genome CDS annotations provided, which was also the case for some of the verified entries as well). As the current species demarcation criterion set by the ICTV Bacterial and Archaeal viruses subcommittee for phages is <95% nucleotide sequence identity, the deduplication of these 13,132 sequences at a 95% sequence identity threshold using cd-hit-est (Li and Godzik, 2006; Fu et al., 2012) resulted in 8,349 genomes that, although most of them have not yet been proposed and/or ratified, represent phage species. On the other hand, 4,783 of the entries were found to be either sequences of the same phage under different accession numbers or phages falling over the current phage species percent identity demarcation criterion. It was noted at this point, that at least 110 out of the deduplicated sequences represented archaeal viruses rather than bacteriophages (Abedon and Murray, 2013) and were, respectively, excluded, leaving 8,239 complete bacteriophage genomes for our phage genome overview. The table was manually refined and *Cystoviridae* phages with segmented genomes missed by the initial filtering step were added, resulting in a total of 8,245 putative phage species genomes.

Taking the latest ICTV BAVS report into account, publicly available complete genomes of viruses of bacteria are currently scattered among at least 19 families (Adriaenssens et al., 2020; ICTV, 2020). Currently, ssDNA bacteriophages (families *Microviridae, Inoviridae, Plectroviridae, Finnlakeviridae)* are represented with 92 putative species complete genomes (∼1.12% of the total genome count), dsDNA phages (*Ackermannviridae, Autographiviridae, Chaseviridae, Demercviridae, Drexleviridae, Herelleviridae, Myoviridae, Podoviridae, Siphoviridae, Sphaerolipoviridae, Corticoviridae, Tectiviridae, Plasmaviridae)* – 7736 genomes (∼93.83%), dsRNA phages (*Cystoviridae)* – 7 genomes (∼0.08%), and ssRNA phages (*Leviviridae)* – 23 genomes (∼0.28%), while the putative phage species unclassified at the family level are represented by 387 genomes corresponding to approximately 4.69% of the total genome number (Table 1).

**TABLE 1:** Overview of completely sequenced phage genomes. Asterix near entry in the column representing the shortest genome of a phage of a given family indicates the shortest plausible entry and ignores ambiguous entries labeled as “complete genome.”

**Family**	**Complete Putative Phage Species Genome Count**	**Percent of the Total Complete Phage Genomes**	**Base pairs**	**Percent of the total base pairs**	**Mean Genome Length ± SD (bp)**	**Phage with the shortest annotated genome/Accession/Genome Length (bp)**	**Phage With the longest annotated genome/Accession/Genome Length (bp)**
*Siphoviridae*	4460	54.09%	230398476	40.43%	51659 ± 23045	Rhodococcus phage RRH1/NC_ 016651.1/14270 bp *	Caulobacter phage CcrBL9/NC_ 048047.1/322272 bp
*Myoviridae*	1608	19.50%	214998333	37.72%	133705 ± 80193	Klebsiella phage ST101-KPC2phi6.2/MK416016.1/11454 bp	Prevotella phage Lak-B8/MK250027.1/551627 bp
*Podoviridae*	571	6.93%	28277096	4.96%	49522 ± 20777	Pectobacterium phage DU_ PP_ III/MF979562.1/11504 bp	Cellulophaga phage phi4:1_ 13/KT962245.1/145865 bp
*Autographiviridae*	481	5.83%	20051636	3.52%	41687 ± 2468	Klebsiella phage PBKP05/MH885472.1/30240 bp	Klebsiella virus 2019KP1/MT360680.1/48372 bp
Unknown family	387	4.69%	7559548	1.33%	19534 ± 23018	Leuconostoc phage L5/L06183.1/2435 bp	Synechococcus phage S-SCSM1/MK867354.1/228827 bp
*Herelleviridae*	219	2.66%	32245234	5.66%	147239 ± 13265	Bacillus phage Maceta/MH538296.1/45023 bp	Bacillus phage AvesoBmore/NC_ 028887.1/167431 bp
*Drexleviridae*	166	2.01%	8034195	1.41%	48399 ± 4451	Escherichia phage IMM-001/MF630922.1/32486 bp *	Klebsiella phage vB_ KpnS_Domnhall/MN013075.1/54438 bp
*Demerecviridae*	120	1.46%	13476523	2.36%	112304 ± 13553	Salmonella phage GE_ vB_ N8/MG969413.1/51134 bp *	Salmonella phage GE_ vB_N5/MG969412.1/148669 bp
*Ackermannviridae*	86	1.04%	13446889	2.36%	156359 ± 4692	Acinetobacter phage SH-Ab 15599/MH517022.1/143204 bp	Ralstonia phage RSP15/NC_ 030948.1/167619 bp
*Inoviridae*	57	0.69%	408238	0.07%	7162 ± 1205	Uncultured phage WW-nAnB/NC_ 026582.1/4817 bp	Vibrio phage CTX/NC_ 015209.1/10638 bp
*Microviridae*	29	0.35%	153147	0.03%	5281 ± 652	Ruegeria phage vB_ RpoMi-V15/MH015251.1/4248 bp	Cellulophaga phage phi12a:1/NC_ 021805.1/6478 bp
*Leviviridae*	23	0.28%	88122	0.02%	3831 ± 365	Enterobacteria phage BZ13 strain T72/FJ483838.1/3393 bp	Enterobacteria phage FI strain BR1/FJ539134.1/4273 bp
*Tectiviridae*	11	0.13%	169814	0.03%	15032 ± 2414	Thermus phage phiKo/MH673671.2/11129 bp	Streptomyces phage WheeHeim/MK305890.1/18266 bp
*Chaseviridae*	7	0.08%	381319	0.07%	54474 ± 1190	Escherichia phage ST32/NC_ 047830.1/53092 bp	Erwinia phage vB_ EamM-Y2/NC_ 019504.1/56621 bp
*Cystoviridae*	7	0.08%	94655	0.02%	13522 ± 715	Pseudomonas phage phi2954/L: NC_ 012091; M: NC_ 012092; S: NC_ 012093/12685 bp	Pseudomonas phage phi8/L: NC_ 003299; M: NC_ 003300; S: NC_ 003301/14984 bp
*Plectroviridae*	5	0.06%	35227	0.01%	7045 ± 1523	Acholeplasma phage MV-L1/NC_ 001341.1/4491 bp	Spiroplasma phage 1-R8A2B/NC_ 001365.1/8273 bp
*Corticoviridae*	4	0.05%	40085	0.01%	10021 ± 831	Marinomonas phage YY/MH105080.1/8828 bp	Vibrio phage fNo16/MH730557.1/10594 bp
*Sphaerolipoviridae* (only Bacterial viruses)	2	0.02%	36640	0.01%	18320 ± 1815	Thermus phage P23-77/NC_ 013197.1/17036 bp	Thermus thermophilus phage IN93/NC_ 004462.2/19604 bp
*Finnlakeviridae*	1	0.01%	9174	0.00%	9174 ± 0	Flavobacterium phage FLiP/NC_047837.1/9174 bp	
*Plasmaviridae*	1	0.01%	11965	0.00%	11965 ± 0	Acholeplasma phage L2/NC_ 001447.1/11965 bp	
**Overall**	8245	100.00%	569916316	100.00%	69163 ± 55772	Leuconostoc phage L5/L06183.1/2435 bp	Prevotella phage Lak-B8/MK250027.1/551627 bp

**FIGURE 1:**
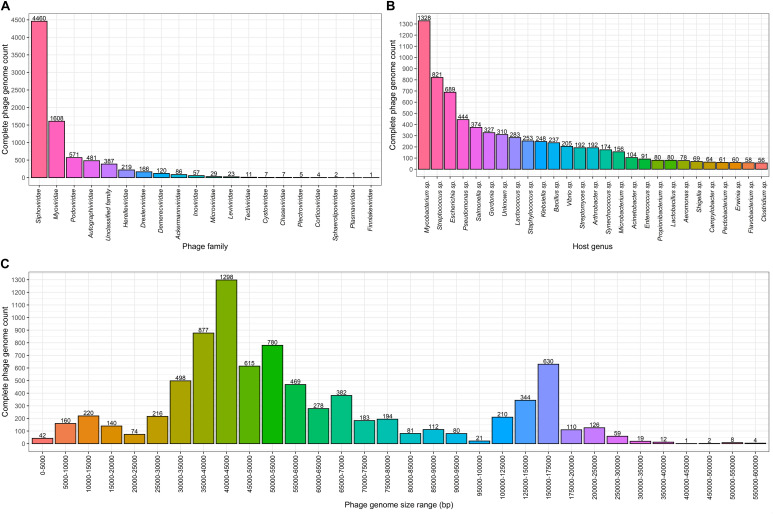
Complete bacteriophage genome count distributions (as of 02.06.2020). Numbers above the bars in each graph indicate complete genome counts (Y) for corresponding X values. **(A)** Completely sequenced bacteriophage genome distribution among viral families where viruses of bacteria are found. The *x* axis represents viral families that include completely sequenced bacteriophage genomes. **(B)** Number of bacteriophages infecting bacterial host genera for which 50 or more infecting bacteriophage complete genomes sequences are available. The *x* axis show bacterial species for which 50 or more corresponding bacteriophages are completely sequenced. **(C)** Size distribution of complete bacteriophage genomes. The *x* axis shows the complete phage genome size ranges (excluding the lowest and including the highest range boundary value): 0–100 kbp with an interval of 5 kbp, 100–200 kbp with an interval of 25 kbp, and 200–600 kbp with an interval of 50 kbp.

Complete genomes of tailed dsDNA-containing phages from the order of *Caudovirales* make up the majority with at least 7,718 out of 8,245 (∼93.6%) complete phage genomes representing different known or yet putative species. It is worth noting that more than half of the complete genomes are those of phages from the *Siphoviridae* family with 4,460 complete genomes that make up ∼54.1% of the total complete phage genome count. The second and third most represented phage families are *Myoviridae* and *Podoviridae*, with 1,608 and 571 completely sequenced genomes, corresponding to ∼19.5 and ∼6.9% of the total completely sequenced phage genome count, respectively (see Figure 1A and Supplementary Figure 1).

Although noticeably lagging behind *Siphoviridae* in terms of complete genome count, 1,608 complete *Myoviridae* phage genomes show a comparable total concatenated genome sequence length to that of the 4,460 *Siphoviridae* phage genomes (40.43% *Sipho-* versus 37.73% *Myo-* of the total complete phage genome sequence length, respectively), which is explained by the fact that the mean complete *Myoviridae* phage genome is 133,705 ± 80,193 bp in length, while the *Siphoviridae* phage genomes are considerably shorter on average (51,659 ± 23,045 bp) (Supplementary Figure 1 and Supplementary Table 1).

Speaking of virion morphotypes, it is worth noting that ICTV BAVS is still reforming the formal bacteriophage taxonomy and have discontinued the use of “morphotype = family” *Caudovirales* taxonomy. For example, while the majority of sipho- phages (long non-contractile tail) are still representatives of *Siphoviridae*, they are now also found in the *Demerecviridae* and *Drexlerviridae* families. Myo- (long contractile tail) and podo- (short non-contractile tail) phages, on the other hand, are currently found in *Myoviridae* and *Podoviridae*, and also in *Ackermannviridae*, *Herelleviridae*, *Chaseviridae* (myophages), and *Autographviridae* (podophages) phage families. With the current rate of novel bacteriophage discovery we can expect that further refinement of the taxonomy is inevitably going to continue and bacteriophage taxonomy is something definitely worth keeping track of.

When comparing complete phage genome counts for phages infecting a particular bacterial genera, *Mycobacterium* phages are the most prominent among phages infecting other bacterial genera, with 1,328 phages that possibly represent a phage species. It is evident within the phage community that this number is a result of the tremendous dedication of the staff involved in “Science Education Alliance Phage Hunters Advancing Genomics and Evolutionary Science (SEA-PHAGES)” and their ability to masterfully involve undergraduates in what becomes their first bacteriophage diversity research endeavor (Jordan et al., 2014). It was noted that 310 (∼3.76%) of the completely sequenced phages do not have their host identified at the genus level. In addition to *Mycobacterium* sp., “TOP5 phage host genera” in terms of completely sequenced corresponding phage genomes include *Streptococcus* sp. with 821 infecting phages, *Escherichia* sp. – 689, *Pseudomonas* sp. – 444, and *Salmonella* sp. – 374, respectively (Figure 1B). At the time of writing, hosts of 7,935 completely sequenced phages (for which hosts have been identified at the genus level) are scattered among only 219 bacterial genera and 74 of these bacterial genera have only a single known completely sequenced phage.

Speaking of length, the most common complete phage genome length still remains in the range of 40–45 kbp, which is consistent with observations made by G. Hatfull in early July 2008, when there were only 500 phage genomes available. The overall distribution has changed substantially with the addition of more than 7,500 additional phage genomes since then (Hatfull, 2008). For example, with the notable growth of the number of representatives in nearly all of the phage genome size ranges, a range of 5–10 kbp is no longer the second most common sequenced phage genome size range (Figure 1C). It can be observed from the genome size distribution plot for putative completely sequenced phage species, that genome sizes of phages seemingly “gravitate” toward three size ranges. Subjectively categorized, “small-sized” (<25 kbp) phage genomes lean toward 10–15 kbp of genome length, “medium-sized” (25–100 kbp) phage genomes – toward 40–45 kbp, and the “large-sized” (>100 kbp) genomes of phages are mostly found within a 150–175 kbp size range (Figure 1C).

## Discussion

Despite the self-explanatory fact that a metaviromics approach might indeed be the fastest way to “mine” potentially useful phage genes from the environmental samples, thus broadening our understanding of the phage pangenome and pinpointing protein candidates for detailed phage-derived product studies, it strictly limits the possibilities of in-depth studies of a particular phage as a microbiological entity (e.g., host range, phage-host interactions, virion stability, and morphology) (Schoenfeld et al., 2010). The amount of data generated during metagenomic studies have pushed phage researchers to develop and constantly improve tools that try to partly overcome some of these difficulties and make *in silico* predictions for some of the aforementioned unknown information purely from the sequencing data, but the positive predictive value of the algorithms used shows that these methods are still hypothetical for most of the novel candidate phage sequences for which no culture is present. This way, based on the sequencing data alone, for example, efforts to predict tailed phage virion morphology (either by the annotation of gene products or using specified classification systems), even if correctly predicting the tail type, give no accurate phage virion dimension estimates (Lopes et al., 2014). Phage host prediction tools, in addition to having a decent accuracy at most, give only an approximate estimation of a host range (Villarroel et al., 2016; Galiez et al., 2017). Genome physical termini predictions (either by prediction tools or manual inspection of reads mapping onto the putative) require a large amount of individual phage reads and may present ambiguous results otherwise (Garneau et al., 2017). Having an individual phage culture on hand, however, despite being harder to acquire, undoubtedly opens up greater research possibilities that might have a more profound impact on the global knowledge of phages than the sequence alone. The question that arises is: can we be sure that the culture-independently acquired putative complete phage genome can be considered “complete” if there is no culturing evidence followed by individual phage genomic nucleic acid acquisition available for experimental verification? Many of the submissions do not have any manuscript linked to it where the methodology would be stated in-detail, and the submission-associated metadata (that are sometimes very scarce) along with the functional annotation are not always enough to evaluate the plausibility of the “complete genome.” This is raising additional concern for metagenomics acquired phage “complete genomes,” evaluation of which should be handled with particular care, possibly including a brief evidence statement on why the submission authors are confident about the “completeness” of the entry in the sequence metadata (e.g., the “circularity” of the assembly).

It was already previously noted by other authors that it is in our interest to better understand the phage phenomena, to not only sequence as many phages as possible, but to also do it for a variety of ecological niches and hosts (Brüssow and Hendrix, 2002; Hatfull, 2015), which is further highlighted by the fact that many of the currently recognized bacterial genera do not yet have the phage that infects its members described (while there is no reason to think that such phages do not exist). Talking about the “breadth” aspect, it is indeed lucrative to shift the phage diversity studies to a metagenomic approach, but there is still a need to broaden the known phage diversity using more traditional culture-based approaches not only, as seen historically, for phages infecting pathogenic bacterial hosts of healthcare and/or economic importance, but also while trying to overcome the great plate count anomaly and hunt for phages of less commonplace host bacteria.

Should the “Hendrix product” (Mushegian, 2020) be the correct estimation of phage particle count on Earth? Taking the horizontal evolution into account, we can expect countless phage species to be described in the near future, and this overview, despite showing where are we in regard to currently available phage diversity, most importantly, signifies that after more than a century since phage discovery we have indeed just begun to uncover phage diversity (Hatfull and Hendrix, 2011) and a plethora of discoveries still lay ahead.

While overviewing the currently available complete phage genomes, some serious yet easily avoidable issues were noted throughout a small number of the submitted and publicly available individual phage complete genome entries, which seemed not to be linked to any particular country, institute, or lab. Taking the rate complete phage genome submission to public repositories into account and addressing concerns about the future usability of entries in such repositories, we, sadly, have to stress the importance of taking the submission process seriously. The excitement of novel phage complete genome acquisition should never obstruct the seriousness with which the authors should treat the public sharing of the data.

First of all, there have been examples of typing errors in the metadata (e.g., “*Escherichia*” or “*Panteoa*” instead of the correct *Escherichia* and *Pantoea*, “Vibro” instead of *Vibrio*, and *Mycobacterium* misspelled in multiple ways). It is highly advisable before submitting a genome to public sequence repositories to re-check the metadata associated with the sequence being deposited without relying solely on the staff of the chosen repository to spot the errors in the metadata of submissions they receive.

Secondly, if the sequence of a phage (bacterial virus) is being submitted, submission authors should try to avoid the ambiguous usage of the sequence-related metadata qualifiers (e.g., “/host = ” qualifier used for organisms other than bacteria); bacteriophages and viruses of bacteria infect and replicate within bacteria, which serve as a natural host. While we advocate for the inclusion of as much genome-related metadata as possible within any submission, we believe that making use of correct sequence-related qualifiers is important, so as not to puzzle other researchers (e.g., if the bacterial host is unknown – stating this in the host field, and adding the higher organism of metagenomic sample to the “/isolation_source = ” qualifier might be more appropriate).

Thirdly, it is unfortunate that, despite there being a wide variety of comparative-genomics based *in silico* genome annotation possibilities, tools, and web-based services offering auto-annotation that require little to no knowledge of bioinformatics, some “complete genomes” were submitted without any annotation of the genomic features of a given phage which strictly limits the possibilities of their further use to other researchers. Physical molecule termini evidence and/or annotation are also often not provided.

Lastly, there should be no “former” submissions, the authors remain responsible for their submissions throughout their whole career. Our understanding of phages grows steadily and, as there will be more known than there is today, we encourage previous contributors to make use of this new knowledge by trying to update their once-submitted phage complete genome annotations every few years. While some researchers might try to “broaden” the known phage diversity, others are steadily working to “deepen” our understanding of phage gene product functions and once hypothetical proteins might already have a function assigned.

## Data Availability Statement

The datasets presented in this study can be found in online repositories. The names of the repository/repositories and accession number(s) can be found in the article/Supplementary Material.

## Author Contributions

NZ summarized and visualized the data and wrote the draft version of the manuscript. AD and AK refined the raw data and identified and fixed the common issues present in the metadata of some complete phage genome entries. AK prepared the final version of the manuscript. All authors conceptualized the study and wrote and edited the manuscript.

## Conflict of Interest

The authors declare that the research was conducted in the absence of any commercial or financial relationships that could be construed as a potential conflict of interest.

## Supplementary Material

Supplementary Figure 1 Fractions of total complete phage genome counts and concatenated sequence length for nine phage families with most “complete genome” representatives. Red bars represent a fraction of the total complete phage genome count. Blue bars represent a fraction of the total complete phage genome concatenated sequence length.

Supplementary Table 1 List of putative phage and archaeal virus species complete genomes.
