# Corrigendum: Motley Crew: Overview of the Currently Available Phage Diversity

**DOI:** 10.3389/fmicb.2020.626744

**Published:** 2021-01-06

**Authors:** Nikita Zrelovs, Andris Dislers, Andris Kazaks

**Affiliations:** Latvian Biomedical Research and Study Centre, Riga, Latvia

**Keywords:** complete genome, phage diversity, bioinformatics, genome overview, bacteriophage

In the original article, there were some errors.

Several corrections have been made in the “Situation as of today” section,

Second paragraph, second sentence:

Currently, ssDNA bacteriophages (families *Microviridae, Inoviridae, Plectroviridae, Finnlakeviridae*) are represented with 92 putative species complete genomes (~1.12% of the total genome count), dsDNA phages (*Ackermannviridae, Autographiviridae, Chaseviridae, Demerecviridae, Drexlerviridae, Herelleviridae, Myoviridae, Podoviridae, Siphoviridae, Sphaerolipoviridae, Corticoviridae, Tectiviridae, Plasmaviridae*) – 7736 genomes (~93.83%), dsRNA phages (*Cystoviridae*) – 7 genomes (~0.08%), and ssRNA phages (*Leviviridae*) – 23 genomes (~0.28%), while the putative phage species unclassified at the family level are represented by 387 genomes corresponding to approximately 4.69% of the total genome number (Table 1).

Fifth paragraph, second sentence:

Myo- (long contractile tail) and podo- (short non-contractile tail) phages, on the other hand, are currently found in *Myoviridae* and *Podoviridae*, and also in *Ackermannviridae, Herelleviridae, Chaseviridae* (myophages), and *Autographiviridae* (podophages) phage families.

Several corrections have been made in the “Discussion” section:

First paragraph, fifth sentence:

Genome physical termini predictions (either by prediction tools or manual inspection of reads mapping onto the putative genome) require a large amount of individual phage reads and may present ambiguous results otherwise (Garneau et al., 2017).

First paragraph, eighth and ninth sentences:

Many of the submissions do not have any manuscript linked to it where the methodology would be stated in-detail, and the submission-associated metadata (that are sometimes very scarce) along with the functional annotation are not always enough to evaluate the plausibility of the “complete genome.” This is raising additional concern for metagenomics acquired phage “complete genomes,” evaluation of which should be handled with particular care, possibly including a brief evidence statement on why the submission authors are confident about the “completeness” of the entry in the sequence metadata (e.g., the “circularity” of the assembly).

Fourth paragraph, second sentence:

Taking the rate of complete phage genome submissions to public sequence repositories into account and addressing concerns about the future usability of entries in such repositories, we, sadly, have to stress the importance of taking the submission process seriously.

Fifth paragraph, first sentence:

First of all, there have been examples of typing errors in the metadata (e.g., “*Eschericha*” or “*Panteoa*” instead of the correct *Escherichia* and *Pantoea*, “*Vibro*” instead of *Vibrio*, and *Mycobacterium* misspelled in multiple ways).

Sixth paragraph, first sentence:

Secondly, if the sequence of a phage (bacterial virus) is being submitted, submission authors should try to avoid the ambiguous usage of the sequence-related metadata qualifiers (e.g., “/host =” qualifier used for organisms other than bacteria); bacteriophages, being viruses of bacteria, infect and replicate within bacteria, which serve as a natural host.

Table 1, column “Family”:

Replace “*Drexlerviridae”* for “*Drexleviridae*”. The corrected Table 1 appears below.

**Table 1:** Overview of completely sequenced phage genomes.

**Family**	**Complete Putative Phage Species Genome Count**	**Percent of the Total Complete Phage Genomes**	**Base pairs**	**Percent of the total base pairs**	**Mean Genome Length ±SD (bp)**	**Phage with the shortest annotated genome/Accession/Genome Length (bp)**	**Phage With the longest annotated genome/Accession/Genome Length (bp)**
*Siphoviridae*	4460	54.09%	230398476	40.43%	51659 ± 23045	Rhodococcus phage RRH1/NC_016651.1/14270 bp *	Caulobacter phage CcrBL9/NC_048047.1/322272 bp
*Myoviridae*	1608	19.50%	214998333	37.72%	133705 ± 80193	Klebsiella phage ST101-KPC2phi6.2/MK416016.1/11454 bp	Prevotella phage Lak-B8/MK250027.1/551627 bp
*Podoviridae*	571	6.93%	28277096	4.96%	49522 ± 20777	Pectobacterium phage DU_PP_III/MF979562.1/11504 bp	Cellulophaga phage phi4:1_13/KT962245.1/145865 bp
*Autographiviridae*	481	5.83%	20051636	3.52%	41687 ± 2468	Klebsiella phage PBKP05/MH885472.1/30240 bp	Klebsiella virus 2019KP1/MT360680.1/48372 bp
Unknown family	387	4.69%	7559548	1.33%	19534 ± 23018	Leuconostoc phage L5/L06183.1/2435 bp	Synechococcus phage S-SCSM1/MK867354.1/228827 bp
*Herelleviridae*	219	2.66%	32245234	5.66%	147239 ± 13265	Bacillus phage Maceta/MH538296.1/45023 bp	Bacillus phage AvesoBmore/NC_028887.1/167431 bp
*Drexlerviridae*	166	2.01%	8034195	1.41%	48399 ± 4451	Escherichia phage IMM-001/MF630922.1/32486 bp *	Klebsiella phage vB_KpnS_Domnhall/MN013075.1/54438 bp
*Demerecviridae*	120	1.46%	13476523	2.36%	112304 ± 13553	Salmonella phage GE_vB_N8/MG969413.1/51134 bp *	Salmonella phage GE_vB_N5/MG969412.1/148669 bp
*Ackermannviridae*	86	1.04%	13446889	2.36%	156359 ± 4692	Acinetobacter phage SH-Ab 15599/MH517022.1/143204 bp	Ralstonia phage RSP15/NC_030948.1/167619 bp
*Inoviridae*	57	0.69%	408238	0.07%	7162 ± 1205	Uncultured phage WW-nAnB/NC_026582.1/4817 bp	Vibrio phage CTX/NC_015209.1/10638 bp
*Microviridae*	29	0.35%	153147	0.03%	5281 ± 652	Ruegeria phage vB_RpoMi-V15/MH015251.1/4248 bp	Cellulophaga phage phi12a:1/NC_021805.1/6478 bp
*Leviviridae*	23	0.28%	88122	0.02%	3831 ± 365	Enterobacteria phage BZ13 strain T72/FJ483838.1/3393 bp	Enterobacteria phage FI strain BR1/FJ539134.1/4273 bp
*Tectiviridae*	11	0.13%	169814	0.03%	15032 ± 2414	Thermus phage phiKo/MH673671.2/11129 bp	Streptomyces phage WheeHeim/MK305890.1/18266 bp
*Chaseviridae*	7	0.08%	381319	0.07%	54474 ± 1190	Escherichia phage ST32/NC_047830.1/53092 bp	Erwinia phage vB_EamM-Y2/NC_019504.1/56621 bp
*Cystoviridae*	7	0.08%	94655	0.02%	13522 ± 715	Pseudomonas phage phi2954/L: NC_012091; M: NC_012092; S: NC_012093/12685 bp	Pseudomonas phage phi8/L: NC_003299; M: NC_003300; S: NC_003301/14984 bp
*Plectroviridae*	5	0.06%	35227	0.01%	7045 ± 1523	Acholeplasma phage MV-L1/NC_001341.1/4491 bp	Spiroplasma phage 1-R8A2B/NC_001365.1/8273 bp
*Corticoviridae*	4	0.05%	40085	0.01%	10021 ± 831	Marinomonas phage YY/MH105080.1/8828 bp	Vibrio phage fNo16/MH730557.1/10594 bp
*Sphaerolipoviridae* (only Bacterial viruses)	2	0.02%	36640	0.01%	18320 ± 1815	Thermus phage P23-77/NC_013197.1/17036 bp	Thermus thermophilus phage IN93/NC_004462.2/19604 bp
*Finnlakeviridae*	1	0.01%	9174	0.00%	9174 ± 0	Flavobacterium phage FLiP/NC_047837.1/9174 bp	
*Plasmaviridae*	1	0.01%	11965	0.00%	11965 ± 0	Acholeplasma phage L2/NC_001447.1/11965 bp	
**Overall**	8245	100.00%	569916316	100.00%	69163 ± 55772	Leuconostoc phage L5/L06183.1/2435 bp	Prevotella phage Lak-B8/MK250027.1/551627 bp

*Asterisk near entry in the column representing the shortest genome of a phage of a given family indicates the shortest plausible entry and ignores ambiguous entries labeled as “complete genome”*.

The authors apologize for all the aforementioned typographic errors and state that they did not change the scientific conclusions of the article in any way.

## References

[B1] Garneau J. R. Depardieu F. Fortier L. C. Bikard D. Monot M. (2017). PhageTerm: A tool for fast and accurate determination of phage termini and packaging mechanism using next-generation sequencing data. Sci. Rep. 7, 1–10. 10.1038/s41598-017-07910-5 28811656 PMC5557969

