# Supplementary material for: Motley Crew: Overview of the Currently Available Phage Diversity
Source: Front Microbiol. 2020 Oct 29;11:579452. doi: 10.3389/fmicb.2020.579452 (PMC7658105; doi:10.3389/fmicb.2020.579452)
Supplement: Supplementary file 2 [file Data_Sheet_1.DOCX]

**Methods**

**Complete phage genome retrieval.** NCBI nucleotide database was queried with the following filter “(viruses[filter] AND (phage[Title] OR (virus[Title] AND gbdiv phg[PROP])) AND biomol_genomic[PROP] AND complete genome[Title])” on 2^nd^ of June 2020 to retrieve only complete bacteriophage genomes in the form of multi entry (13132 entries) *.gb and multifasta files (Sayers et al., 2019).

**Complete phage genome nucleotide sequence deduplication.** Multifasta file containing 13132 retrieved sequences was deduplicated at 95% sequence identity threshold using cd-hit-est (Li and Godzik, 2006; Fu et al., 2012). Duplicate entries were discarded from the multi entry *gb file based on their accession numbers.

**Data curation and correction.** Locus, Genome length, Title, Accession number, Taxonomy, Host, Lab host, CDS count, Viral Family, Host genus, and whether retrieved sequence is a phage of archaeal virus were retrieved for each locus of multi entry GenBank file containing deduplicated sequences using regular expression based in-house Python 3 scripts written for the task to form a tab delimited table. Table was manually checked for ambiguous entries in the extracted information, typos made by authors of original submissions were corrected. Lack of phage genomes belonging to family *Cystoviridae* (for each species of which complete genome is represented by three individual dsRNA segment sequences) was noted and they were subsequently manually included in a manner that provides accessions to each of the segments but sums CDS count and genome length from all of the three individual *Cystoviridae* phage genome segments to represent complete genome. The final table representing an easily searchable snapshot of the complete bacteriophage genome diversity for phages that could be putatively recognized as species was used for further overview (provided as Supplementary Table 1). All the manipulations during this step were performed in Microsoft Excel.

**Figure generation.** The resulting table from the previous step was imported in R version 3.5.1 (2018-07-02) as a data frame and used for necessary manipulations to generate barplots using R package ggplot2 (Wickham, 2009).

**References**

Sayers, E. W., Cavanaugh, M., Clark, K., Ostell, J., Pruitt, K. D., and Karsch-Mizrachi, I. (2019). GenBank. *Nucleic Acids Res.* 47, D94–D99. doi:10.1093/nar/gky989.

Li, W., and Godzik, A. (2006). Cd-hit: A fast program for clustering and comparing large sets of protein or nucleotide sequences. *Bioinformatics* 22, 1658–1659. doi:10.1093/bioinformatics/btl158.

Fu, L., Niu, B., Zhu, Z., Wu, S., and Li, W. (2012). CD-HIT: Accelerated for clustering the next-generation sequencing data. *Bioinformatics* 28, 3150–3152. doi:10.1093/bioinformatics/bts565.

Wickham, H. (2009). *Ggplot2: Elegant Graphics for Data Analysis*. 2nd ed. Springer Publishing Company, Incorporated.
